# CORRELATION OF SHOULDER RANGE OF MOTION WITH LOWER EXTREMITY PERFORMANCE AMONG COMMUNITY-DWELLING OLDER ADULTS

**DOI:** 10.1093/geroni/igad104.3251

**Published:** 2023-12-21

**Authors:** Ranyah Almardawi, Vicki Gray, Nathan Frakes, Yuanyuan Liang, Derik Davis

**Affiliations:** University of Maryland School of Medicine, Baltimore, Maryland, United States; University of Maryland School of Medicine, Baltimore, Maryland, United States; University of Maryland School of Medicine, Baltimore, Maryland, United States; University of Maryland School of Medicine, Baltimore, Maryland, United States; University of Maryland School of Medicine, Baltimore, Maryland, United States

## Abstract

More than one-third of older adults have mobility limitations. Yet the impact of shoulder dysfunction on lower extremity performance is only nascent in description. We tested the hypothesis that shoulder range of motion (ROM) correlates with lower extremity performance. Data were collected from 26 community-dwelling older women (80.8%) and men aged ≥ 65 years (mean±standard deviation=71.9±5.3 years) enrolled in a pilot study at one time point. Bilateral shoulder ROM testing for forward flexion (FF), abduction (ABD), and adducted external rotation (Add-ER); and Short Physical Performance Battery (SPPB), gait speed (GS), 6-meter narrow walk test (6M-NWT), and stair climb power test (SCPT) were performed. The sum of bilateral FF, ABD, and Add-ER was determined (Total-ROM) for each participant. Spearman’s rank-order correlation (rho) with 95% confidence intervals (CIs) was used to measure the strength and direction of correlation for Total-ROM with the mobility outcome measures. The study sample demonstrated Total-ROM, 709.5° ± 80.1°; SPPB score, 10.9 ± 1.3; GS, 1.2 ± 0.2 m/s, 6M-NWT time, 7.3 ± 3.9 s; and SCPT, 240.7 ± 90.7 watts. Total-ROM correlated with: SPPB 0.56 (0.22,0.78), p=0.003; GS, 0.59 (0.27,0.80), p=0.001; SCPT, 0.54 (0.20,0.77), p=0.004; and 6M-NWT, -0.63 (-0.82,-0.33), p< 0.001. The results provided preliminary evidence that shoulder ROM correlates moderately with measures of lower extremity performance in community-dwelling older adults. Future studies are warranted in older populations to test for the longitudinal association of shoulder ROM with lower extremity performance and to determine if treatment of shoulder dysfunction provides effective prehabilitation to mitigate age-related declines in mobility.

